# Recent Advances and Future Directions in Bellafill as a Regenerative Biomaterial: Biological Mechanisms, Clinical Applications, and Safety

**DOI:** 10.1111/jocd.70923

**Published:** 2026-06-12

**Authors:** Li Nan, Fang Xia, Ahmad Alhaskawi, Chenggang Yi, Pengchao Guo

**Affiliations:** ^1^ Department of Plastic Surgery The Second Affiliated Hospital of Zhejiang University School of Medicine Hangzhou China; ^2^ Department of Orthopedics, the First Affiliated Hospital Zhejiang University Hangzhou Zhejiang Province P. R. China; ^3^ Emergency Department The Second Affiliated Hospital of Zhejiang University School of Medicine Hangzhou China

**Keywords:** ArteFill, Bellafill, collagen neogenesis, injectable biomaterials, polymethyl methacrylate, scaffold‐based regeneration, soft‐tissue regeneration

## Abstract

**Background:**

Bellafill is a polymethylmethacrylate (PMMA) microsphere‐collagen composite increasingly recognized as a scaffold‐based injectable biomaterial rather than a temporary filler. Its non‐resorbable PMMA microspheres enable stable integration into host tissue and promote collagen neogenesis, fibroblast activation, and extracellular matrix remodeling while maintaining favorable long‐term biocompatibility.

**Methods:**

This narrative review synthesizes clinical and translational evidence retrieved from PubMed and Web of Science to evaluate Bellafill's biological mechanisms, safety profile, and expanding clinical applications.

**Results:**

Clinical studies support its efficacy in atrophic acne scars, dermal atrophy, and soft‐tissue augmentation across diverse patient populations. Emerging off‐label applications in wound healing and reconstructive settings further highlight its regenerative potential.

**Conclusion:**

Collectively, current evidence positions Bellafill as a regenerative injectable platform that extends beyond aesthetic volumization and warrants further investigation in soft‐tissue regeneration.

## 1 Introduction

Soft‐tissue defects arising from aging, trauma, inflammation, infection, or congenital conditions remain a persistent clinical challenge due to the limited intrinsic regenerative capacity of adult connective tissues. Conventional reconstructive strategies, including surgical grafting and temporary injectable fillers, primarily focus on volume replacement rather than restoration of native tissue architecture and function [1, 2, 3]. In recent years, regenerative medicine has shifted toward biomaterial‐based approaches that actively guide host cell behavior, promote extracellular matrix (ECM) remodeling, and achieve long‐term tissue integration rather than transient correction [4, 5]. Injectable biomaterials have emerged as minimally invasive regenerative platforms capable of delivering structural support while engaging endogenous repair mechanisms [6]. However, most widely used injectable fillers, particularly biodegradable materials such as hyaluronic acid, provide only short‐term volumization and are rapidly degraded, necessitating repeated interventions and offering limited regenerative benefit [7, 8]. This limitation has prompted growing interest in scaffold‐based injectable biomaterials that can sustain tissue remodeling and biological replacement over extended periods.

**FIGURE 1:**
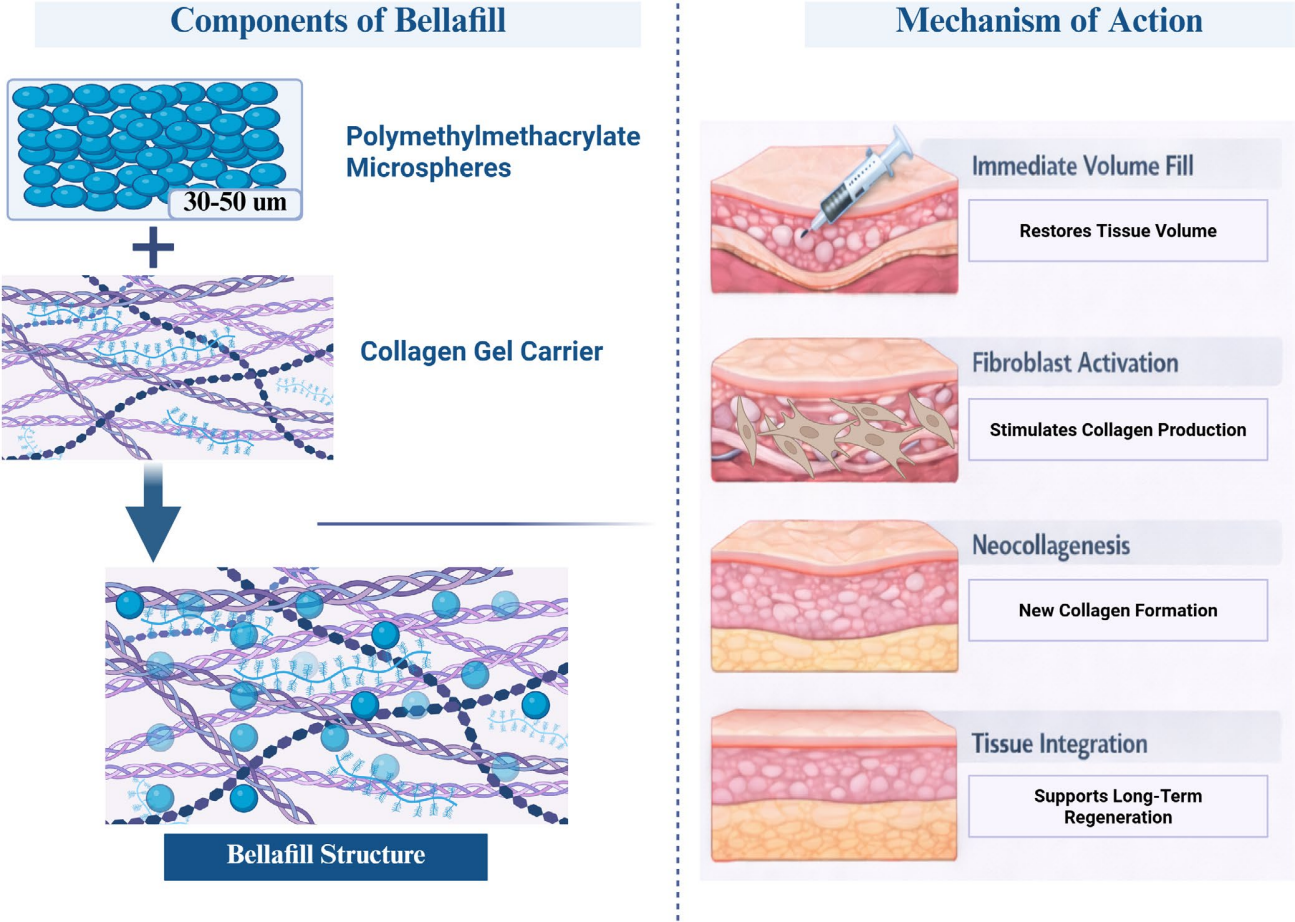
Bellafill composition and mechanism of action.

Bellafill, an FDA‐approved dermal filler composed of polymethylmethacrylate (PMMA) microspheres suspended in a bovine collagen carrier, has been extensively employed for long‐lasting aesthetic correction of nasolabial folds and acne scars (Figure 1). Its unique biostimulatory profile, primarily through the induction of neocollagenesis, has prompted interest in its broader clinical applications [9, 10]. Unlike transient fillers, Bellafill is designed for long‐term tissue integration, owing to the non‐resorbable nature of PMMA microspheres, which serve as a scaffold for sustained fibroblast activity and ECM remodeling [11, 12]. The regenerative properties observed in aesthetic treatments have led to preliminary investigations into Bellafill's utility in different therapeutic settings. These off‐label applications represent a promising frontier but remain underexplored in the biomedical literature. Therefore, this narrative review aims to synthesize current knowledge on the clinical use of Bellafill as a regenerative biomaterial. In addition, it seeks to clarify the material's biological mechanisms, assess its translational potential, and identify research gaps that warrant further investigation.

## 2 Method

A narrative literature review was conducted to summarize recent clinical and translational evidence on Bellafill and polymethyl methacrylate (PMMA), based on injectable biomaterials. A structured literature search was performed across multiple biomedical and scientific literature sources. Search terms included “Bellafill,” “ArteFill,” “PMMA,” “polymethyl methacrylate,” “injectable biomaterial,” “collagen neogenesis,” and “soft‐tissue regeneration.” Original clinical studies, histological investigations, reviews, and translational biomaterials research relevant to scaffold‐based tissue remodeling were considered. Non‐PMMA fillers and reports lacking clinical or biological relevance were excluded. The literature was qualitatively summarized to highlight material characteristics, biological mechanisms, and clinical applications of Bellafill from a regenerative biomaterial perspective.

### 2.1 Biocompatibility and Immune Response

**FIGURE 2:**
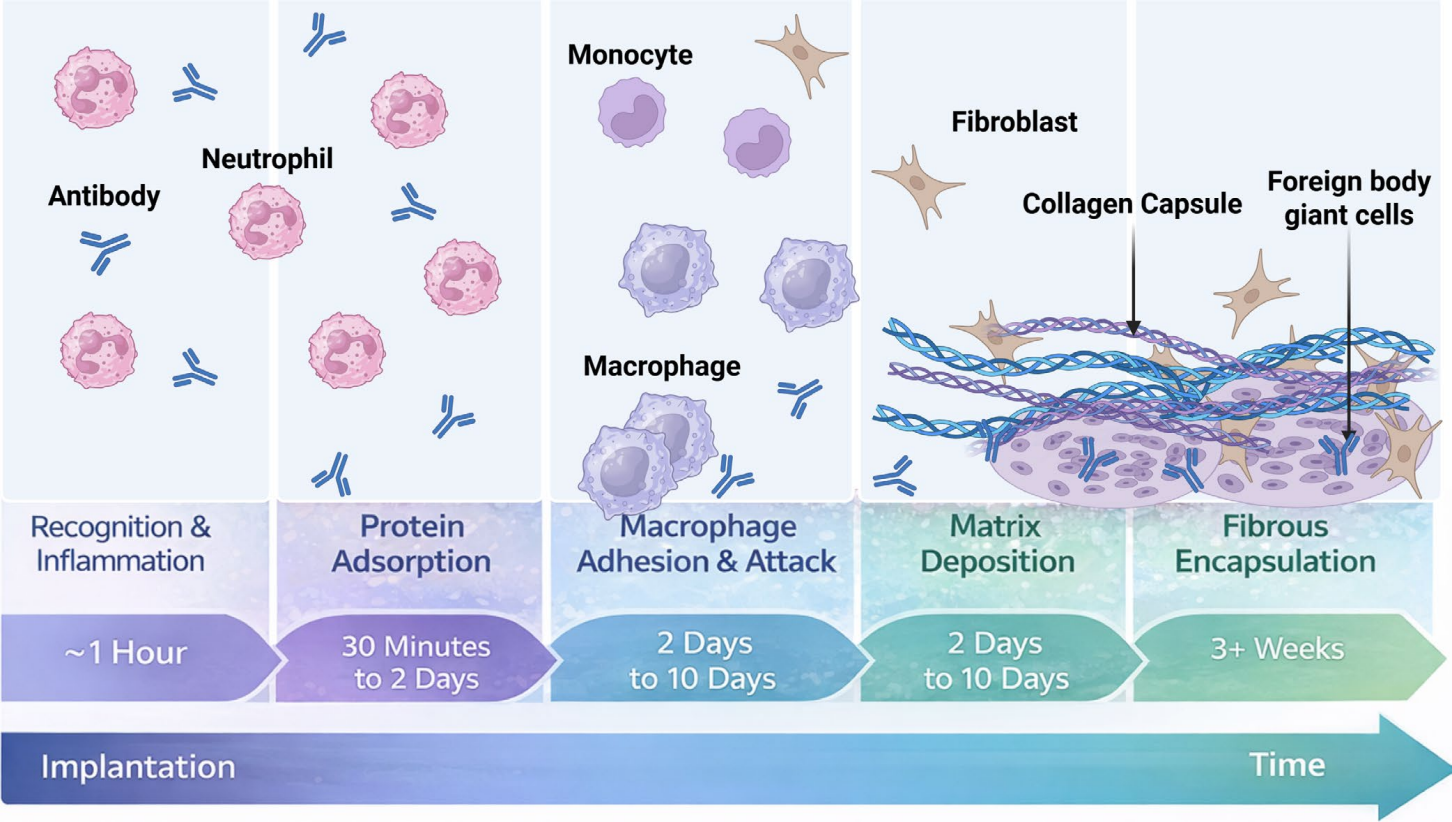
Host tissue response to an implanted biomaterial over time.

Bellafill consists of uniformly sized polymethylmethacrylate microspheres embedded within a collagen‐based matrix, formulated with lidocaine to enhance procedural comfort. The long‐term clinical success of PMMA fillers like ArteFill is underpinned by their carefully engineered biocompatibility and their ability to modulate the host's immune response [13, 14]. At the forefront of this design is the avoidance of unwanted inflammatory or granulomatous reactions that plagued earlier PMMA formulations. The microspheres in ArteFill are smooth, round, and uniformly sized (30–50 μm), a range deliberately selected to avoid phagocytosis by macrophages, which typically engulf particles below 10–15 μm in diameter (Figure 1) [15, 16]. Following injection into the dermis or subdermis, the host tissue initiates a controlled acute immune response. Neutrophils are the first responders, infiltrating the site within hours to days. Their presence is transient and serves to clear any incidental tissue debris or contaminants introduced during injection. Notably, studies have shown that neutrophil accumulation is minimal with ArteFill due to the sterile, non‐immunogenic nature of the material and its carrier collagen [16, 17, 18]. Macrophages arrive shortly after and attempt to survey the microspheres. Because of the PMMA particles' smooth surface and large size, macrophages are unable to engulf them. Instead of initiating a classical pro‐inflammatory response, the macrophages enter a regulatory or wound‐healing phenotype (often referred to as M2‐like polarization). This phenotype supports tissue remodeling and fibrous encapsulation rather than inflammation or necrosis (Figure 2) [19, 20, 21]. Histological evaluations confirm the absence of typical inflammatory markers such as lymphocytic infiltration, necrosis, or active degradation pathways [19, 20, 21, 22]. Over time, a foreign body‐type reaction develops, not in the pathologic sense but as a stable integration process. Multinucleated giant cells may form at the surface of some microspheres, a hallmark of foreign body reaction [23]. However, this response is limited and remains non‐destructive. The PMMA microspheres are gradually surrounded by fibroblasts, collagen fibers (notably collagen type I and III), and new capillaries, forming a biocompatible, vascularized scaffold that mirrors the characteristics of normal connective tissue [11, 24]. This structured encapsulation plays a vital role in the material's permanence and stability. Unlike earlier generations of PMMA fillers (e.g., Arteplast, which included non‐uniform and small particles), ArteFill's refined microsphere architecture prevents clustering, migration, and excessive fibrotic capsule formation, all of which had previously contributed to nodules or granulomas (Table 1) [9, 15, 25].

**TABLE 1:** Comparative characteristics of PMMA fillers [15].

Feature	Arteplast	Artecoll	Artefill	Bellafill
Development stage	First generation	Second generation	Third generation	Optimized third generation
Era of introduction	Late 1980s	1990s	Early 2000s	2015 (rebranded)
Carrier material	Gelatin	Bovine collagen	Bovine collagen & lidocaine	Bovine collagen & lidocaine
Microsphere size	20–40 μm, variable	More uniform	Highly uniform (30–50 μm)	Highly uniform (30–50 μm)
Small‐particle contamination	Common (1–5 μm)	Greatly reduced	< 1% < 20 μm	< 1% < 20 μm
Surface characteristics	Irregular, charged	Smooth, uncharged	Smooth, round	Smooth, round
Recommended injection plane	Intradermal	Deep dermis/subdermal	Deep dermis/dermal‐subcutaneous junction	Deep dermis/dermal‐subcutaneous junction
Granuloma incidence	Relatively high (~2%–3%)	Very low (~0.01%–0.02%)	Low	Very low (≈0.002% per syringe post‐marketing)
FDA approval (USA)	No	No	Yes (nasolabial folds)	Yes (nasolabial folds, acne scars)
Long‐term evidence‐based	Limited	Moderate	Strong	Extensive (clinical trials + ≥ 10‐year surveillance)
Current clinical relevance	Historical	Limited	Superseded	Widely used, FDA‐approved

Histologically, at around 3 months post‐injection, microspheres are embedded within dense collagen networks, with active fibroblasts and newly formed vessels present throughout the matrix. By 6–12 months, the implanted area becomes a composite of approximately 80% host‐derived collagenous tissue and 20% PMMA microspheres [26]. Importantly, the collagen formed is not a scar‐like, disorganized mass but rather a well‐integrated ECM that supports dermal integrity and aesthetics [13]. Over time, the PMMA microspheres remain intact, stable, and fully integrated into the connective tissue without signs of chronic inflammation, migration, or resorption, and no degradation or systemic dissemination of microspheres will occur [27, 28]. These characteristics have raised interest in its use not only for cosmetic volume replacement but also in contexts where durable soft tissue support and regenerative stimulation are desired, such as wound healing, dermal atrophy, and surgical site augmentation. Importantly, while PMMA microspheres themselves are biologically inert, their physical presence in the tissue induces a mechanotransductive effect that appears to activate local cells and remodel the microenvironment over time.

### 2.2 Clinical Applications

While Bellafill has been widely utilized for cosmetic enhancement, particularly in the treatment of nasolabial folds and atrophic acne scars, emerging evidence suggests that its unique material characteristics and biostimulatory properties may extend its utility into therapeutic and regenerative domains [9]. These off‐label applications remain in early stages of exploration but reflect a growing interest in harnessing Bellafill as more than a volume‐restoring filler.

#### 2.2.1 Acne Scars and Dermal Atrophy

Atrophic acne scars and dermal atrophy are the result of chronic inflammation‐induced degradation of dermal collagen and ECM, leading to permanent structural deficits and surface depression of the skin. Despite the availability of multiple therapeutic approaches, most existing interventions focus on temporary volume replacement or superficial resurfacing, with limited capacity to induce true dermal regeneration [29]. In this context, Bellafill has emerged as a regenerative treatment strategy, as its persistent PMMA microspheres extend beyond simple filling to stimulate collagen remodeling and progressive dermal thickening, thereby addressing the underlying structural deficiency of atrophic scars. Clinical trials have demonstrated that these effects can be durable for up to 5 years, even after the initial bovine collagen carrier is resorbed [11]. Histopathological analysis has confirmed dense collagen encapsulation around PMMA microspheres, with minimal inflammatory infiltrate. Notably, collagen remodeling extends beyond the immediate site of injection, suggesting broader dermal restructuring, a hallmark of regenerative activity. Patients with dermal atrophy from chronic inflammatory conditions may similarly benefit from this biostimulatory effect, although formal studies in these populations remain limited [11, 30]. Joseph et al. found that Bellafill significantly improved moderate‐to‐severe atrophic facial acne scars, achieving a 64.4% responder rate at 6 months compared with 32.6% in saline controls, with high patient satisfaction and no treatment‐related serious adverse events, supporting both its clinical efficacy and safety for long‐term acne scar correction [10]. Another study reported that full‐face treatment of distensible atrophic acne scars with Bellafill resulted in high clinical efficacy and patient satisfaction, with > 90% of subjects achieving ≥ 1‐grade improvement on the Acne Scar Assessment Scale at 4 and 7 months, alongside minimal injection volumes and no serious treatment‐related adverse events, supporting the short‐term safety and effectiveness of Bellafill beyond cheek‐limited applications [31]. In addition, a double‐blind, randomized multicenter trial showed that Bellafill significantly improved atrophic facial acne scars, with 64% treatment success versus 33% in controls, early onset of benefit, and mild, transient adverse events, with efficacy consistent across age, sex, and skin phototypes, including darker skin types, supporting its efficacy and short‐term safety for acne scar correction [32]. Together, these studies show that Bellafill provides significant improvement of atrophic acne scars through sustained collagen remodeling, with consistent efficacy and a favorable safety profile, supporting its role as a long‐term regenerative treatment rather than a temporary filler.

#### 2.2.2 Wound Healing and Soft Tissue Regeneration

Wound healing is a dynamic and highly regulated biological process involving hemostasis, inflammation, proliferation, and remodeling phases aimed at restoring tissue integrity [33]. In parallel, soft tissue regeneration focuses not only on closure but on the restoration of functional ECM and vascular structures. Fibroblast activation, collagen deposition, angiogenesis, and immune modulation are all critical components of effective repair. Disruptions in these processes, due to ischemia, infection, or systemic conditions, can result in chronic, non‐healing wounds that are refractory to standard treatments [34, 35]. Recent regenerative strategies incorporate biomaterial scaffolds, growth factors, and cell‐based therapies to recreate the native tissue environment and promote endogenous healing responses [36]. The use of Bellafill in wound healing and soft tissue regeneration has emerged as a promising off‐label application, particularly in cases involving chronic wounds, post‐surgical depressions, and soft tissue atrophy from trauma or disease [10, 37]. Preliminary case reports and small clinical series suggest that Bellafill acts as a structural scaffold that supports tissue repair. Its formulation provides immediate mechanical support while facilitating fibroblast migration, neocollagenesis, and matrix remodeling. This dual mechanism may help stabilize wound architecture and promote tissue integration, making Bellafill a potentially valuable adjunct in complex reconstructive settings. A recent case report highlighted the use of Bellafill as a collagen carrier for topical delivery of a purified exosome product (PEP) in the treatment of chronic, nonhealing scalp wounds following chemoradiation and surgical reconstruction. Application of Bellafill‐based exosome therapy was associated with complete closure of a frontal scalp defect and a 96% reduction in a temporoparietal wound, with no treatment‐related adverse events, underscoring the potential role of Bellafill as an effective scaffold supporting regenerative wound healing in refractory cases [38].

#### 2.2.3 Perioral and Periorbital Rejuvenation

**FIGURE 3:**
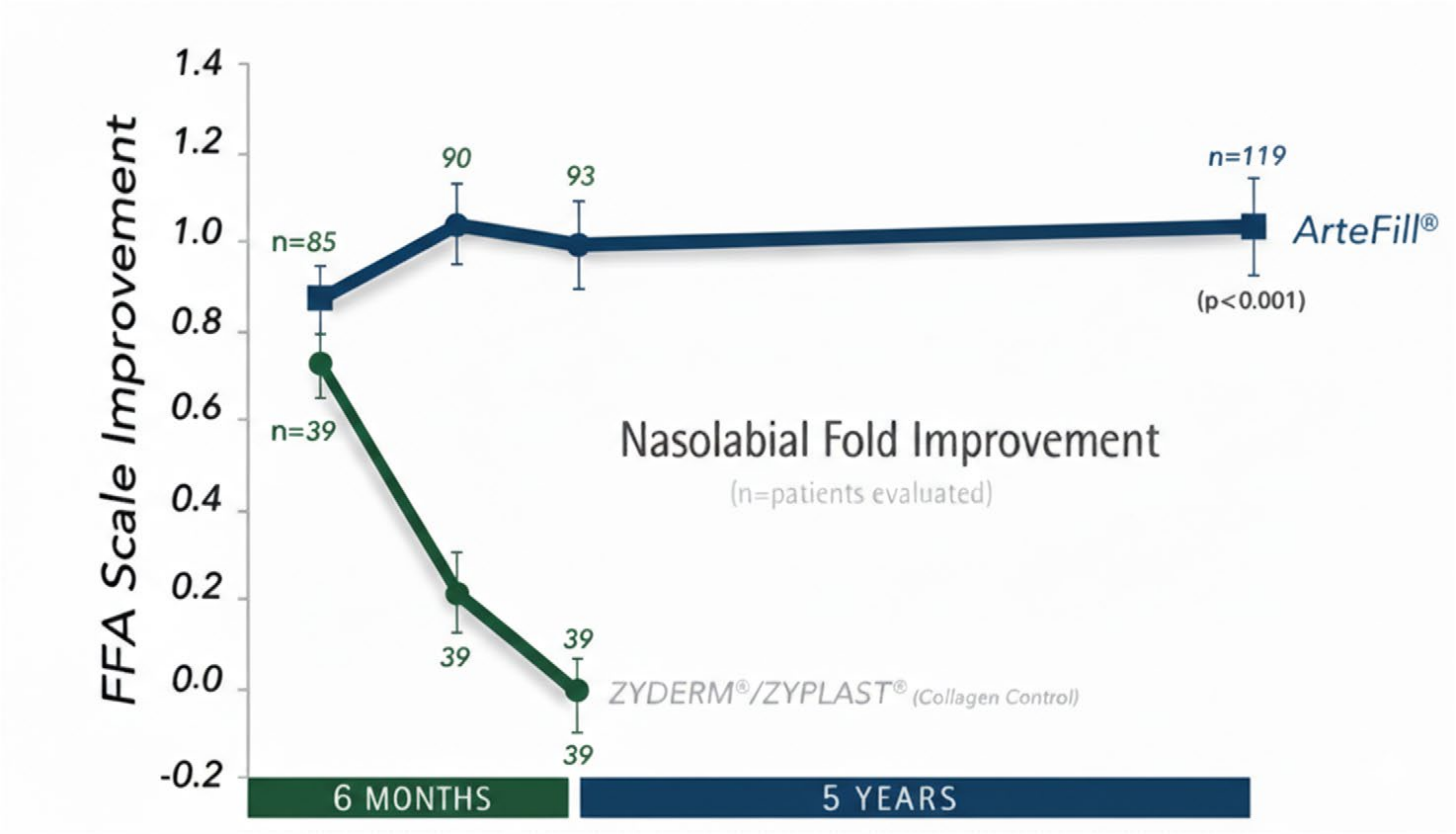
Long‐term nasolabial fold improvement following ArteFill injection [45].

Age‐related soft tissue degeneration around the mouth and eyes is often marked by loss of dermal integrity, fine wrinkling, and volume collapse, all of which can be partially addressed by biostimulatory fillers [39, 40]. Bellafill has shown considerable efficacy in rejuvenating these complex regions, where structural reinforcement is required rather than just superficial plumping. The mechanical tension induced by the microspheres can initiate mechanotransduction pathways, stimulating fibroblast proliferation and promoting dermal thickening. Over time, these effects improve tissue resilience and elasticity, with some reports noting improved skin tone and texture well beyond the initial treatment zone. Furthermore, these outcomes point to true dermal regeneration, suggesting that Bellafill could serve as a minimally invasive tool for reversing localized atrophic changes in elderly or photodamaged skin, applications that are fundamentally regenerative, despite being categorized under cosmetic procedures [10]. Lemperle et al. described a novel off‐label use of Bellafill for treating senile enophthalmos‐associated epiphora by restoring age‐related loss of intraorbital volume through retrobulbar injection. By advancing the receded globe and re‐establishing eyelid‐conjunctival apposition, the procedure eliminated the lateral canthal “wind‐trap,” leading to immediate resolution of refractory outdoor tearing, widening of the palpebral fissure by approximately 2 mm, and sustained functional and aesthetic improvement for up to 12 months, with imaging confirming stable filler localization [41]. Different studies investigated non‐hyaluronic acid fillers for midface augmentation, reporting that Bellafill achieves durable malar volumization with high patient and physician satisfaction, supported by prospective clinical evidence demonstrating sustained improvement at 12 months and a favorable short‐term safety profile. The authors emphasize that, due to its permanent collagen‐stimulating properties, Bellafill requires careful patient selection, conservative volume use, and experienced injection technique [42, 43]. In addition, a case report demonstrated that Bellafill can provide durable, nonsurgical correction of lower‐face asymmetry caused by idiopathic masseter muscle hypoplasia. In a young adult patient unwilling to undergo surgery, staged subcutaneous injections of Bellafill along the mandibular border achieved marked and sustained facial symmetry, with high patient satisfaction and no short‐ or long‐term complications observed during 1‐year follow‐up. This report highlights the role of Bellafill as a long‐lasting volumetric solution in selected patients requiring stable correction where temporary fillers or surgery are suboptimal [44]. A study found that ArteFill achieved sustained improvement in nasolabial folds, with facial fold assessment scale scores remaining stable from 6 months to 5 years after injection, whereas collagen‐based fillers showed a rapid decline in efficacy within the first year (Figure 3) [45].

### 2.3 Safety, Immunogenicity, and Long‐Term Outcomes

Bellafill is distinguished by its permanence and long‐term integration within soft tissue. While these properties contribute to its regenerative potential, they also raise legitimate concerns about long‐term biocompatibility, immune response, and the risk of late‐onset adverse events, including granuloma formation. The current body of evidence supports a generally favorable safety profile for Bellafill, though vigilance in patient selection, technique, and post‐treatment monitoring remains essential [10]. In a large 5‐year prospective safety and satisfaction study, Cohen et al. followed over 1000 patients receiving Bellafill for nasolabial fold correction and found a low incidence of adverse events, with confirmed granuloma formation reported in fewer than 1% of treated subjects. Notably, most adverse effects, including lumps, nodules, or swelling, occurred within the first year post‐injection and were typically localized, manageable, and non‐systemic [46]. Along with that, a comprehensive study by Paulucci analyzed PMMA fillers and granuloma incidence, concluding that Bellafill's granuloma rates ranged from 0.01% to 0.4%, considerably lower than earlier PMMA formulations like Artecoll and Arteplast, which had higher impurity levels and inconsistent microsphere sizing, both factors now mitigated by Bellafill's strict particle uniformity and collagen carrier filtration [47]. From an immunological standpoint, Bellafill demonstrates low immunogenicity due to the inertness of PMMA and the highly purified bovine collagen carrier. Nonetheless, hypersensitivity reactions have been occasionally documented, particularly in patients with a prior history of bovine collagen allergy or autoimmune conditions. For this reason, a pre‐treatment skin test is required to identify collagen hypersensitivity, although the occurrence of true allergic reactions remains exceedingly rare [48]. Gold et al. found that Bellafill demonstrated a strong safety and efficacy profile over a 10‐year postmarketing period, with extremely low rates of adverse events, including granulomas (0.002% of over 530 000 syringes distributed). The evolution of Bellafill from earlier PMMA fillers like Arteplast and Artecoll involved significant improvements in microsphere uniformity and purification techniques, which greatly reduced complications [15, 49]. Furthermore, Solomon et al. found that Bellafill demonstrated a low adverse event rate (1.4%) across 417 procedures in 212 patients, with no granulomas reported over a 4‐year clinical period. The study observed high patient satisfaction, especially for acne scar correction (99%) and rhinoplasty touch‐ups (96%), with lower satisfaction noted in high‐volume areas like jaw angles [50]. A prospective in vivo study showed that Bellafill remains structurally intact and histologically unaffected following treatment with a wide range of laser, light, and ultrasound energy‐based modalities, with no abnormal tissue reactions or adverse events observed. These findings support the safety of combining Bellafill with energy‐based aesthetic treatments [51]. However, expert consensus continues to emphasize the importance of injection technique, specifically deep dermal or subdermal placement, small volume per pass, and avoidance of superficial injection, which is more likely to result in visible irregularities or nodularity. Patients with active inflammatory skin disease, unrealistic expectations, or autoimmune disorders are considered relatively contraindicated for Bellafill use [48] (see Table 2).

**TABLE 2:** Safety, immunogenicity, and long‐term outcomes of Bellafill.

Domain	Parameter	Characteristics	Clinical evidence/notes
Safety [46, 47, 50]	Early adverse events	Mild, transient reactions (erythema, swelling, tenderness); predominantly within first weeks	Self‐limiting; no systemic toxicity reported
	Nodules/irregularities	Rare; mainly technique‐related (superficial placement or excessive volume)	Reduced by deep dermal/subdermal injection and conservative dosing
	Granuloma formation	Very low incidence (≈0.002%–0.4%)	Markedly lower than first‐ and second‐generation PMMA fillers
	Long‐term complications	No evidence of migration, degradation, or systemic dissemination	PMMA microspheres remain structurally intact
Immunogenicity [48, 52, 53]	Acute inflammatory response	Minimal and transient neutrophil infiltration	Consistent with controlled wound‐healing response
	Macrophage response	Predominantly regulatory (M2‐like) phenotype	Supports remodeling rather than chronic inflammation
	Foreign body reaction	Stable, non‐pathologic foreign body encapsulation	Multinucleated giant cells may be present but non‐destructive
	Allergic reactions	Extremely rare; linked to bovine collagen sensitivity	Pre‐treatment skin testing recommended
Histological Integration [10, 11]	Microsphere behavior	Non‐phagocytosable, smooth, round PMMA particles	Prevents macrophage engulfment and migration
	Collagen neogenesis	Progressive deposition of collagen type I and III	~80% host collagen/~20% PMMA at 3 months
	Vascularization	Neovascular ingrowth within collagen matrix	Indicates functional tissue integration
Long‐Term Outcomes [10, 15, 50, 51]	Durability of correction	Clinical efficacy sustained ≥ 5–10 years	Stable wrinkle and acne scar improvement
	Structural stability	No volume loss after carrier collagen resorption	PMMA scaffold maintains tissue architecture
	Patient satisfaction	High satisfaction rates (> 90% in acne scar studies)	Consistent across age, sex, and skin phototypes
	Compatibility with devices	Safe with laser, light, and ultrasound therapies	No histologic or clinical adverse interactions

## 3 Future Perspectives

Although Bellafill is already supported by substantial long‐term clinical experience, its biological behavior as a scaffold‐based injectable biomaterial suggests several important directions for future research and clinical translation. From a regenerative medicine perspective, further mechanistic studies are needed to elucidate the cellular and molecular pathways governing PMMA‐induced tissue remodeling, particularly fibroblast phenotypic modulation, macrophage polarization, and long‐term ECM organization [54]. Advanced histological and single‐cell analyses may provide deeper insight into how permanent microscale scaffolds interact with host tissues over extended timeframes. Another promising avenue lies in the integration of Bellafill with biologically active therapies. Combination strategies involving platelet‐rich plasma, growth factor delivery, or cell‐derived secretomes could potentially enhance early regenerative responses while preserving the long‐term structural benefits of the PMMA scaffold [55, 56]. Such hybrid approaches may allow fine‐tuning of early inflammation, angiogenesis, and matrix deposition, thereby expanding regenerative indications beyond current clinical use. From a materials science standpoint, future innovations may focus on next‐generation PMMA‐based injectable scaffolds, including surface‐modified microspheres, composite carriers, or controlled‐release systems designed to modulate host responses without compromising material stability. These advances could further optimize biocompatibility, spatial tissue integration, and regenerative efficiency. Clinically, expanding applications into reconstructive and post‐traumatic soft‐tissue repair represents an important translational opportunity. Conditions characterized by chronic volume loss, matrix degradation, or poor intrinsic healing may benefit from permanent scaffold‐mediated stabilization. However, these applications require carefully designed prospective studies to define indications, safety profiles, and outcome measures beyond aesthetic endpoints.

Finally, long‐term imaging modalities and quantitative tissue assessment tools should be incorporated into future clinical studies to objectively evaluate tissue remodeling, collagen architecture, and biomechanical properties over time. Such data will be essential for repositioning Bellafill and related PMMA‐based systems more definitively within the field of regenerative biomaterials rather than cosmetic fillers.

## 4 Conclusion

Bellafill represents a stable injectable scaffold with the capacity to support long‐term soft‐tissue integrity when used appropriately. Ongoing advances in biomaterial design and clinical technique highlight its potential relevance beyond cosmetic use, warranting further investigation within regenerative soft‐tissue reconstruction.

## Author Contributions

Writing – original draft: L.N., and F.X.; Writing – review and editing: A.A., P.G. and C.Y.; Validation: P.G., L.N., and F.X.; Data collection: A.A., and C.Y.; Supervision: P.G., and A.A.

## Funding

The authors have nothing to report.

## Ethics Statement

The authors have nothing to report.

## Consent

The authors have nothing to report.

## Conflicts of Interest

The authors declare no conflicts of interest.

## Data Availability

The authors have nothing to report.
